# Supplementary material for: Modelling Skylarks (Alauda arvensis) to Predict Impacts of Changes in Land Management and Policy: Development and Testing of an Agent-Based Model
Source: PLoS One. 2013 Jun 6;8(6):e65803. doi: 10.1371/journal.pone.0065803 (PMC3675089; doi:10.1371/journal.pone.0065803)
Supplement: Supporting Information S4 — The skylark ODdox as a zipped archive. (ZIP) [file pone.0065803.s004.zip › Skylark_ODdox/struct_e_g_p___data.html]

ALMaSS Skylark ODdox: EGP\_Data Struct Reference


ALMaSS Skylark ODdox
 2.0


- Main Page
- Related Pages
- Classes
- Files

- Class List
- Class Index
- Class Hierarchy
- Class Members

Public Attributes

EGP\_Data Struct Reference

`#include <CIPELandscapeMaker.h>`

List of all members.

|  |  |
| --- | --- |
| Public Attributes | |
| int | m\_cx |
| int | m\_cy |
| int | m\_orig\_size |
| int | m\_polynum |

---

## Member Data Documentation

int EGP\_Data::m\_cx

int EGP\_Data::m\_cy

int EGP\_Data::m\_orig\_size

int EGP\_Data::m\_polynum

---

The documentation for this struct was generated from the following file:

- CIPELandscapeMaker.h


- EGP\_Data
- Generated on Thu Jan 10 2013 13:15:36 for ALMaSS Skylark ODdox by
   1.8.1.1
